# Structure Modification Converts the Hepatotoxic Tacrine into Novel Hepatoprotective Analogs

**DOI:** 10.1021/acsomega.3c07126

**Published:** 2024-01-02

**Authors:** Amani A. Sorour, Rania G. Aly, Hanan M. Ragab, Ahmed Wahid

**Affiliations:** † Department of Pharmaceutical Biochemistry, Faculty of Pharmacy, Alexandria University, Alexandria 21521, Egypt; ‡ Department of Pathology, Faculty of Medicine, Alexandria University, Alexandria 21521, Egypt; § Department of Pharmaceutical Chemistry, Faculty of Pharmacy, Alexandria University, Alexandria 21521, Egypt

## Abstract

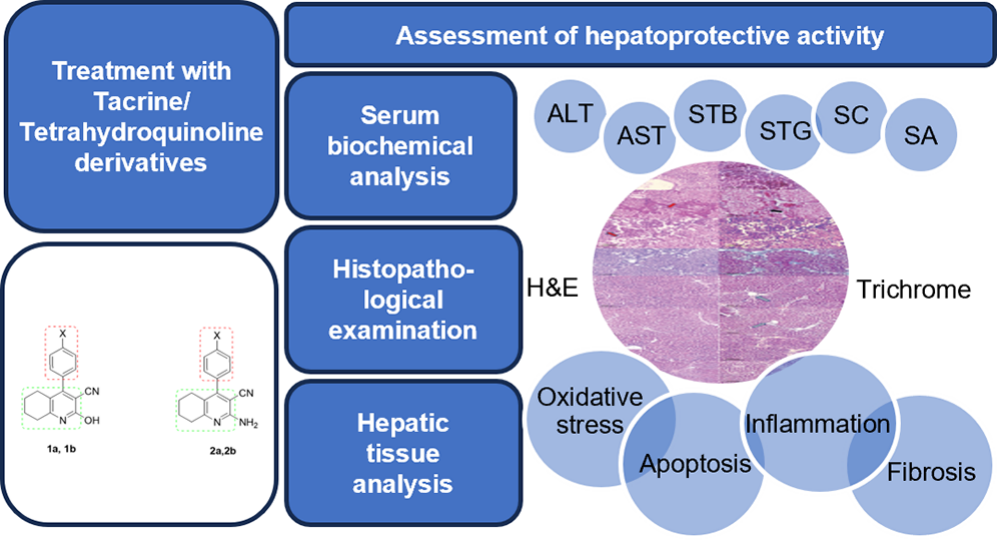

The liver is responsible for critical functions such as metabolism, secretion, storage, detoxification, and the excretion of various compounds. However, there is currently no approved drug treatment for liver fibrosis. Hence, this study aimed to explore the potential hepatoprotective effects of chlorinated and nonchlorinated 4-phenyl-tetrahydroquinoline derivatives. Originally developed as tacrine analogs with reduced hepatotoxicity, these compounds not only lacked hepatotoxicity but also displayed a remarkable hepatoprotective effect. Treatment with these derivatives notably prevented the chemically induced elevation of hepatic indicators associated with liver injury. Additionally, the compounds restored the activities of defense antioxidant enzymes as well as levels of inflammatory markers (TNF-α and IL-6), apoptotic proteins (Bax and Bcl2), and fibrogenic mediators (α-SMA and TGF-β) to normal levels. Histopathologic analysis confirmed the hepatoprotective activity of tetrahydroquinolines. Furthermore, computer-assisted simulation docking results were highly consistent with those of the observed in vivo activities. In conclusion, the designed tacrine analogs exhibited a hepatoprotective role in acute liver damage, possibly through their antioxidative, anti-inflammatory, and antifibrotic effects.

## 1 Introduction

The liver is a crucial organ in the human body and is essential for homeostatic maintenance.^1^ Most hepatotoxic substances destroy hepatocytes, increasing tissue lipid peroxidation and oxidative stress.^2^ Hepatic fibrosis is associated with apoptosis, leading to excess extracellular matrix (ECM) deposition.^3,4^ Hepatic stellate cell (HSC) activation is a primary driver of ECM protein secretion.^3,4^ Activated HSCs are proliferative and fibrogenic, rapidly producing ECM components such as transforming growth factor-beta (TGF-β), tumor necrosis factor-alpha (TNF-α),^3,4^ and apoptotic proteins (Bax and Bcl2).

Liver injury leads to fibrosis, causing specific changes in all of the liver cells. Studies show that activated HSCs contribute to ECM degradation through oxidative stress via reactive oxygen species (ROS), leukocyte chemotaxis through chemokine and cytokine production, and hepatocyte growth factor (HGF) production. Oxidative stress plays a role in the development of liver fibrosis.^5^ The injured liver can have various sources of oxidative stress, one of which is the cytochrome P450 2E1 enzyme that is found in hepatocytes. Infiltrating lymphocytes contribute to inflammation. The inflammatory response of hepatocytes is essential for the progression of hepatic fibrosis. Hepatocytes undergo injury and programmed cell death (apoptosis). Quiescent stellate cells are activated and produce ECM proteins, forming the fibrous scar.^6^ When activated, HSCs lose retinoids, express new receptors such as transforming growth factor-β (TGF-β) receptors, and produce new proteins like α receptors and actin. They also proliferate and synthesize ECM proteins.^7^

**Figure 1:**
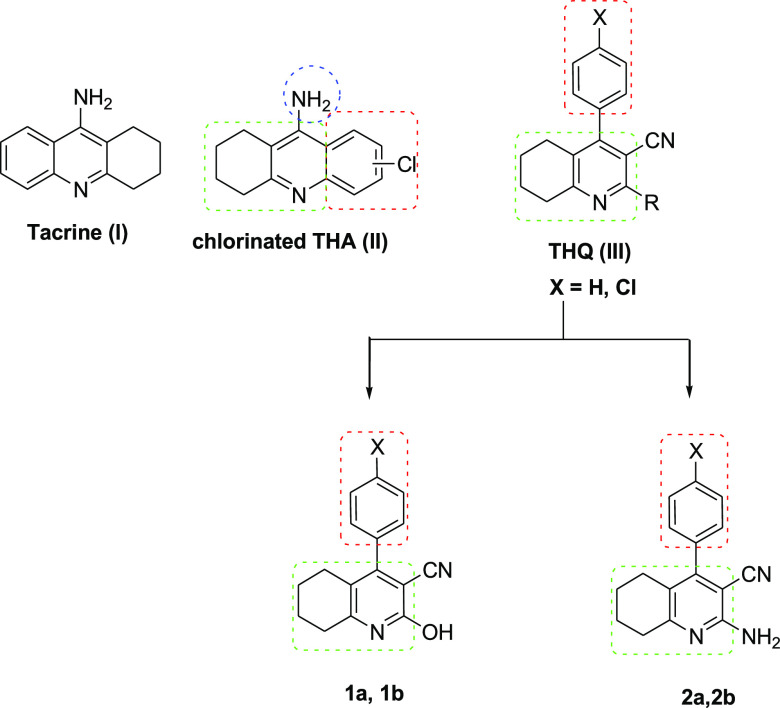
Chemical structure of tacrine (I), chlorinated tacrine (II), 4-aryltetrahydroquinoline (THQ; III) as a basic nucleus for the target compounds **1a**, **1b**, **2a**, and **2b**. The chemical structures of compounds **1a** and **1b** include hydroxide and cyanide substitutes, while compounds **2a** and **2b** include amino and cyanide substitutes. Letter **b** refers to the chloride group.

The compounds tested in this study were not primarily designed with the aim of reversing liver injury. They were initially designed in a previous study conducted in our laboratory to inhibit the cholinesterase enzyme for Alzheimer’s disease treatment. This former study was based on the structural modification of tacrine (compound I, Figure 1), which is a reversible, noncompetitive acetylcholinesterase inhibitor (AChEI) known to possess hepatotoxic side effects.^8,9^ Accordingly, the design was aimed at modifying tacrine to produce potent molecules that had minimal hepatotoxicity.

Tacrine-induced acute hepatotoxicity has been extensively studied in the literature.^8,10,11^ Watkins et al.,^8^ showed that individuals who were administered tacrine had levels of alanine transaminase (ALT) higher than normal. Lou et al.^11,12^ found that tacrine caused considerable liver impairments. However, there has been less research on structural modifications of tacrine that may lead to minimizing toxicity.^13,14^ Reddy et al. designed several tacrine derivatives, with lower hepatotoxicity than those for tacrine.^13^ Quintanova et al. also designed hybrid-tacrine substances with favorable neuroprotective properties.^14^

Accordingly, the design of compounds used in our previous study aimed at splitting the tetrahydroacridine (THA) moiety present in tacrine into two portions: a tetrahydroquinoline nucleus and a phenyl ring. The separated phenyl ring was introduced at the 4-position of the tetrahydroquinoline moiety, resulting in compounds (III, where X = H, Figure 1) that proved to be highly effective as AChEIs.^15,16^ Furthermore, the insertion of a chloro substituent at the 4-phenyl ring has been previously shown to significantly improve the cholinesterase activity (compound II, Figure 1).^17^ Combining both of these modifications resulted in (compound III, where X = Cl, Figure 1) improving cholinesterase inhibitory activity. Interestingly, these compounds demonstrated hepatoprotective effects in an experimental model of acute liver injury induced by the administration of DMF, lowering the ALT level.^15^

The results of our former study warranted a follow-up study to further investigate these compounds as hepatoprotective agents. We chose to assess the chlorinated and nonchlorinated 4-phenyl-3-cyano-2-substituted hydroxy (**1a** and **1b**) and amino (**2a** and **2b**) tetrahydroquinolines (Figure 1). The testing protocol involved the induction of hepatic injury using carbon tetrachloride (CCl_4_) in rats,^18^ which leads to progressive tissue injury, with inflammation being the initial indicator.^18,19^ Afterward, the capability of the compounds under investigation to reverse this injury was assessed relative to silymarin.^20^ Silymarin is a flavonoid derived from *Silybum marianum* seeds^21^ that possesses potent antioxidant activities, limiting lipid peroxidation and cell membrane damage.^22^

The novelty of this study lies in the exploration of a detailed mechanistic study of the potential hepatoprotective activity of the previously synthesized THQ derivatives. The compounds were initially designed and synthesized in our laboratory as acetylcholine esterase inhibitors with reduced hepatotoxicity. However, the exact mechanism of the hepatoprotection of the compounds was not investigated. Accordingly, the target of this study was to clarify and elaborate on the hepatoprotective mechanism of action of these compounds by studying different liver injury pathways, including oxidative stress, inflammation, apoptosis, and fibrosis.

## 2 Results

### 2.1 Toxicity Study

#### 2.1.1 Effect of THQ Derivatives on Rats’ Vitals and Body Weight

**Figure 2:**
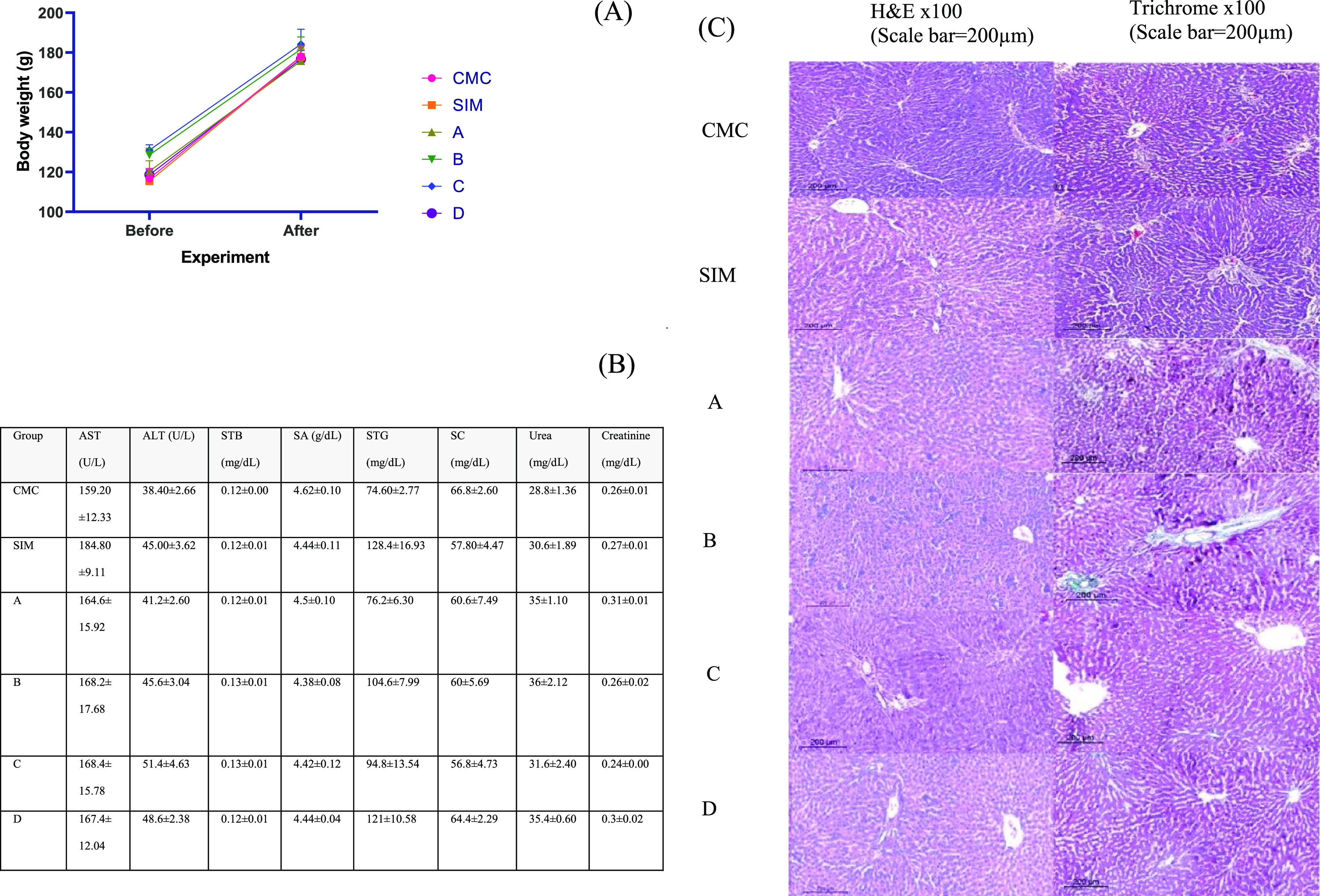
(A) Male albino rats’ body weight through the 14 days of the experiment. At a 5% level of significance (*p < 0.05), no significant difference was obvious between the groups; *n* = 5; (B) biochemical parameters of the toxicity study for the synthetic drugs (A: **1a**, B: **1b**, C: **2a**, and D: **2b**); (C) effect of THQ-derivatives on liver histology. It showed no pathological effect on liver tissue as represented by hematoxylin and eosin and Masson Trichrome; values are expressed in mean ± SEM (*n* = 5).

The toxic reactions observed in preclinical models are crucial in determining the safety profile of substances with potential pharmacological effects. In our investigation, all rats were monitored daily to check for any toxicity symptoms after the treatment. Each rat’s body weight was measured before and after the experiment. All treated rats survived the 14 day experiment, with no mortality being recorded during the experimental period. No toxicity indications were detected in the rats’ vitals during the observation period. Compounds **(**A: **1a**, B: **1b**, C: **2a**, and D: **2b)** had no toxic effects on the animals’ body weight; hence, there was no noticeable difference between the control and treated groups, and all the animals showed a normal increase in body weight (Figure 2).

#### 2.1.2 Hepatic and Renal Safety of the Compounds

The assessment of the toxicity of the synthesized compounds on Wistar rats is shown in Figure 2. The levels of hepatic biomarkers (ALT, AST, and total bilirubin; STB and albumin; and SA), lipid biomarkers (Triglycerides; STG and total cholesterol levels; and SC), and renal biomarkers (urea and creatinine) were examined in the serum. The results indicated that both silymarin and THQ derivatives exhibited similar liver, renal, and lipid profiles as those in the carboxy methyl cellulose (CMC) group.

#### 2.1.3 A, B, C, and D Preserved Normal Cellular Architecture of the Liver

Histological analysis was performed to further characterize any alteration in the structure of the liver at the cellular level. In this study, evaluation of liver architecture and necrosis by using different staining protocols (hematoxylin and Eosin and Masson’s trichrome) was performed. The histopathological examination revealed normal liver parenchyma and portal tract architecture, with no evidence of steatosis, ballooning, inflammation, or fibrosis in CMC-treated rats as well as both Silymarin and THQ derivatives-treated rats. It showed the general appearance of classic hepatic lobules formed of the central vein and peripheral portal tracts. Cords of hepatocytes were seen radiating from the central. Hepatocytes appeared polygonal in shape with acidophilic cytoplasm and central, rounded vesicular nuclei. Portal tracts contained bile ducts, branches from the hepatic artery, and portal veins (Figure 2).

### 2.2 Therapeutic Model Treated after the Induction of Fibrosis

#### 2.2.1 THQ Derivatives Improved the Serum Biological Parameters of CCl_4_-Treated Rats

The hepatic effects of the synthetic compounds employed in the current study on CCl_4_-intoxicated rats are tabulated in Table.1. In the CCl_4_-treated group, the levels of AST, ALT, STB, STG, and SC liver biomarkers were increased, while those of SA decreased. Treatment with synthetic drugs (A: **1a**, B: **1b**, C: **2a**, and D: **2b**) significantly mitigated the increase in ALT, AST, STB, STG, and SC levels and significantly elevated SA to a level close to normal values. Therefore, treatments under investigation induced a significant improvement in the level of basic liver parameters compared with those in the group treated with silymarin, a known hepatoprotective agent, and those in the healthy control group.

**Table 1:** Effect of the Synthetic Drugs on CCl_4_-Induced Hepatotoxicity in Wistar Albino Rats; AST, ALT, STB, SA, STG, and SC^a^

group	treatment	AST (U/L)	ALT (U/L)	STB (mg/dL)	SA (g/dL)	STG (mg/dL)	SC (mg/dL)
control	corn oil (1 mL/kg)	180.00 ± 8.94	47.20 ± 1.83	0.12 ± 0.00	4.8 ± 0.05	90.80 ± 3.84	63.80 ± 0.86
model	CCl_4_ (1 mL/kg)	807.60 ± 47.85^####^	528.40 ± 27.12^####^	0.24 ± 0.05^##^	3.68 ± 0.08^####^	105.60 ± 12.14	85.40 ± 1.89^####^
CCl_4_ + CMC	CCl_4_ (1 mL/kg) + CMC (3 mL/kg)	654.20 ± 18.82^####^	428.20 ± 27.99^####^	0.23 ± 0.02^##^	3.94 ± 0.10^####^	118.60 ± 7.88	82.80 ± 1.39^####^
CCl_4_ + SIM	CCl_4_ (1mL/Kg) + silymarin (100 mg/Kg)	686.00 ± 91.28	419.00 ± 77.57	0.18 ± 0.02	4.26 ± 0.07****	174.80 ± 14.52****	91.60 ± 2.48
CCl_4_ + A	CCl_4_ (1 mL/kg) + drug **1a** (25 mg/kg)	178.35 ± 10.54****	72.80 ± 6.74****	0.15 ± 0.01*	4.58 ± 0.04****	96.20 ± 1.50	72.20 ± 1.46**
CCl_4_ + B	CCl_4_ (1 mL/kg) + drug **1b** (25 mg/kg)	162.60 ± 13.44****	49.40 ± 1.96****	0.13 ± 0.01**	4.58 ± 0.05****	100.00 ± 2.98	72.40 ± 1.5**
CCl_4_ + C	CCl_4_ (1 mL/kg) + drug **2a** (25 mg/kg)	182.40 ± 16.06****	62.00 ± 4.28****	0.13 ± 0.01**	4.6 ± 0.08****	104.60 ± 7.24	72.80 ± 1.46**
CCl_4_ + D	CCl_4_ (1 mL/kg) + drug **2b** (25 mg/kg)	181.00 ± 13.42****	43.40 ± 4.37****	0.12 ± 0.01**	4.46 ± 0.09****	90.00 ± 8.33	70 ± 4.49***

a Values are expressed as mean ± S.E.M, (*n* = 5). *P*-value: 0.0332(*), 0.0021(**), 0.0002(***), and >0.0001(****). Statistical analysis was performed using a one-way ANOVA followed by the Tukey–Kramer multiple comparisons test. # Significantly different from the normal control group, * Significantly different from the model group, and + Significantly different from the CCl_4_ + CMC control group.

#### 2.2.2 THQs Derivatives Alleviated Collagen Deposition in Liver Tissues in Comparison to the CCl_4_-Treated Rats

**Figure 3:**
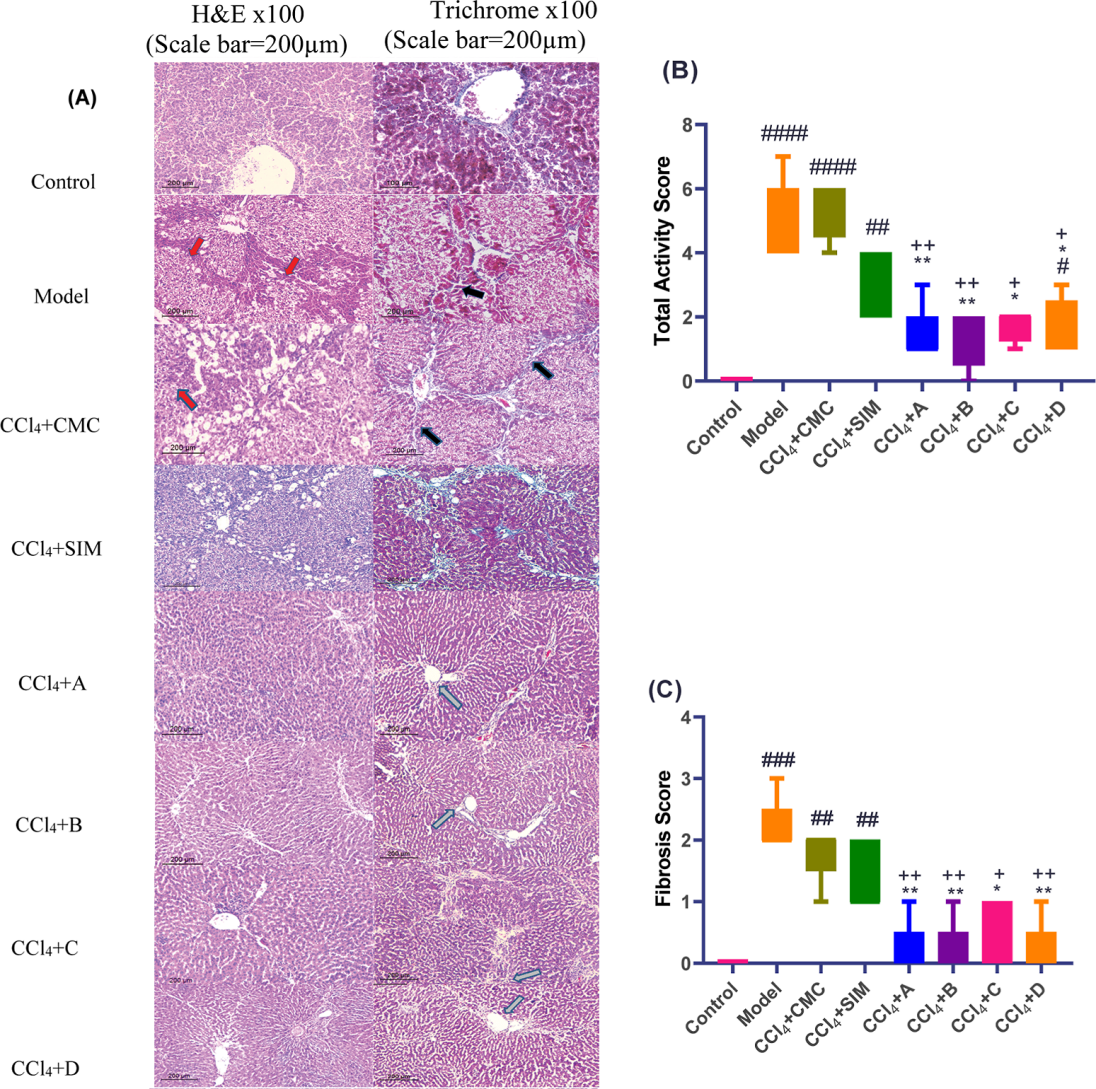
(A) Microscopic analysis of liver tissue. The model group showed severe steatosis, lobular inflammation, ballooning of the hepatocytes, and extensive bridging fibrosis in the liver. THQ-Derivatives showed variable degrees of restoration of hepatic architecture as represented by hematoxylin and eosin, Masson Trichrome (Red arrow: steatosis, blue arrow: lobular inflammation, and black arrow: bridging fibrosis); (B) histological scoring of steatohepatitis (steatosis (0–3), lobular inflammation (0–2), and hepatocellular ballooning (0–2)); (C) staging of fibrosis (0–4). Bars represent the mean ± SEM (*n* = 5). *P*-value: 0.0332(*), 0.0021(**), 0.0002(***), and >0.0001(****). Statistical analysis was performed using Kruskal–Wallis’s test, followed by Dunn’s multiple comparisons test. #Significantly different from the normal control group, *significantly different from the model group, and +significantly different from the CCl_4_ + CMC control group.

Liver histology for control animals showed normal liver parenchyma with preserved cytoplasm, a prominent nucleus and nucleolus, and a portal tract. In comparison, the CCl_4_ and CCl_4_ + CMC.-treated groups showed severe steatosis, lobular inflammation, ballooning of the hepatocytes, and extensive bridging fibrosis in the liver. The histopathological liver architecture following treatment with drugs A, B, and D showed normal liver architecture and a portal tract, whereas animals treated with drug C showed normal liver architecture and a portal tract with focal mild steatosis. The silymarin-treated group showed moderate steatosis and focal bridging fibrosis. In all treated groups, histopathologic examination revealed normal liver parenchyma and portal tract architecture, with no evidence of steatosis, ballooning, inflammation, or fibrosis (Figure 3).

#### 2.2.3 Antioxidant Properties of A, B, C, and D in Reducing Hepatic Fibrosis

**Figure 4:**
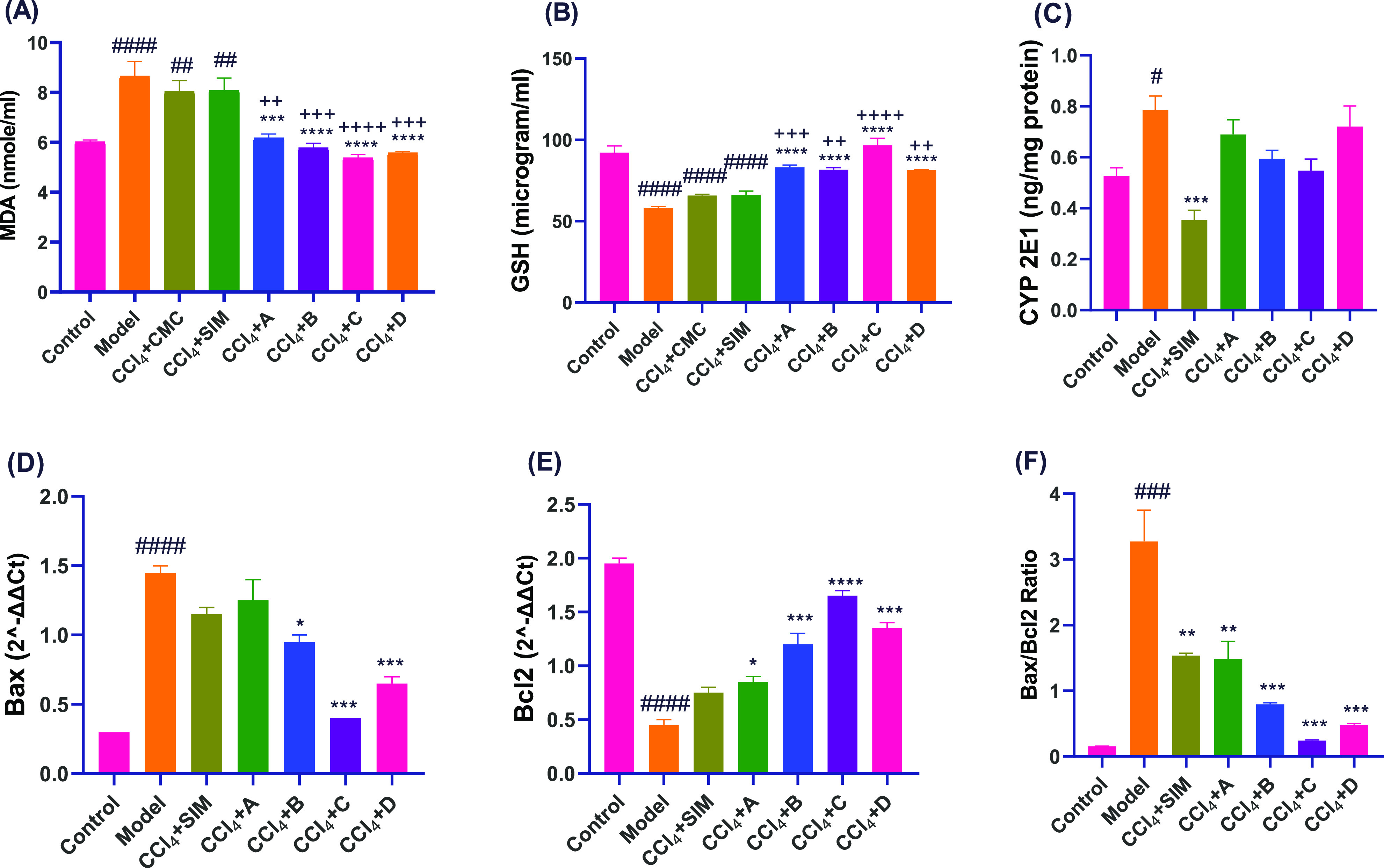
Oxidative stress markers in liver tissue homogenate such as MDA (A) and GSH(B). Bars represent mean ± SEM (*n* = 5); (C) CYP 2E1 protein expression values by ELISA analysis. Bars represent mean ± SEM (*n* = 3). M-RNA expression by RT-PCR analysis of (D) Bax, (E) Bcl2, and (F) Bax/Bcl2 ratio values. Bars represent the mean ± SEM (*n* = 2). *P*-value: 0.0332(*), 0.0021(**), 0.0002(***), and >0.0001(****). Statistical analysis was performed using a one-way ANOVA followed by the Tukey–Kramer multiple comparisons test. #Significantly different from the normal control group, *Significantly different from the model group, + Significantly different from the CCl_4_ + CMC control group.

As oxidative stress is one of the pathological processes linked to the development of fibrosis, we examined the antioxidant effects of THQ derivatives. In the model group, CCl_4_-induced liver injury elevated the level of CYP2E1 protein expression and liver malondialdehyde (MDA) and caused a reduction in liver glutathione (GSH) activity compared with those in the healthy control group. Our results revealed that THQ derivative treatment decreased CYP2E1 protein expression, albeit without statistical significance, dramatically decreased liver MDA levels, and markedly increased the activity of GSH compared to the CCl_4_ group (Figure 4).

#### 2.2.4 THQs Derivatives Enhanced the Apoptosis Regulators of CCl_4_-Treated Rats

In liver fibrosis, apoptosis plays a critical role in the initiation and propagation of liver injury to liver fibrosis. Expression of the proapoptotic Bax gene was downregulated in the groups treated with the four drugs; however, this was statistically significant only with drugs B, C, and D. Reverse transcription polymerase chain reaction (RT-PCR) analysis showed that there was a significant upregulation in the expression of the antiapoptotic Bcl2 mRNA following treatment with drugs A, B, C, and D, resulting in a decrease in the Bax/Bcl2 ratio, as shown in Figure 4.

#### 2.2.5 THQs Exert an Anti-Inflammatory Effect on CCl_4_-Treated Rats

**Figure 5:**
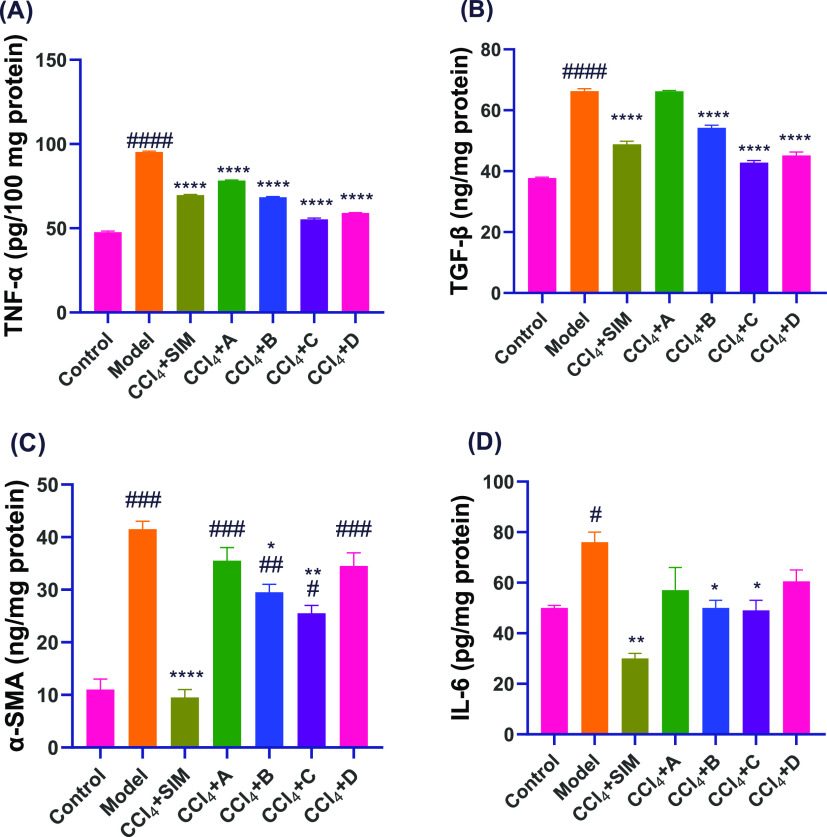
Proinflammatory and profibrogenic cytokines level of expression through fibrosis and after treatment; (A) TNF- α and (B) TGF- β, Bars represent the mean ± SEM (*n* = 3). (C) α -SMA and (D) IL-6 protein expression levels by ELISA analysis. Bars represent the mean ± SEM (*n* = 2). *P*-value: 0.0332(*), 0.0021(**), 0.0002(***), and >0.0001(****). Statistical analysis was performed using a one-way ANOVA followed by the Tukey–Kramer multiple comparisons test. #Significantly different from the normal control group, and *significantly different from the model group.

Inflammation is a central feature of liver fibrosis. We used the levels of TNF-α and IL-6 protein expression as an index of the severity of inflammation. CCl_4_ injection caused an increase in the expression of the pro-inflammatory cytokines (TNF-α and IL-6). THQs successfully ameliorated this increase and restored the expression to normal levels (Figure 5).

#### 2.2.6 THQs Derivatives Exhibited an Antifibrotic Effect in CCl_4_-Treated Rats

Regression of fibrosis is characterized by a decline in α-SMA and TGF-β. Levels of α-SMA and TGF-β were elevated in the livers of CCl_4_-treated rats. Surprisingly, administration of THQ derivatives reduced the expression levels of these markers toward a normal level (Figure 5).

### 2.3 In-Silico Prediction of the D1–3 Possible Mechanism of Action

Docking analysis using crystal structures revealed that compounds **1b**, **2a**, and **2b** showed superior binding to the Bax receptor, where all three drug compounds formed three hydrogen bonds with the receptor, whereas compounds **1a** and silymarin had inferior binding with only one hydrogen bond formed with the receptor. These results were consistent with the above-mentioned in vivo results (see Supporting Information Figure 1).

However, the binding mode to Bcl2 indicated superior binding for compounds **2a** and **2b** with two hydrogen bonds forming with the receptor versus only one hydrogen bond formed by compound **1b** and arene–H bonding between compound **1a** and the receptor (see Supporting Information Figure 2).

Analysis of binding to IL-6 showed that compounds **2a** and **2b** again had superior binding, forming three hydrogen bonds with the receptor, while compound **1a** only formed one hydrogen bond with an arene-cation binding to the receptor, whereas only hydrophobic binding was predicted between compound **1b** and the receptor (see Supporting Information Figure 3).

For TGF-β, the binding of compound **2a** was superior to all the tested compounds with two hydrogen bonds present, while only one was formed with compound **2b**; compound **1a** formed two arene–H bonds, and compound **1b** showed only one arene–H bond to the receptor (see Supporting Information Figure 4).

Finally, TNF-α binding with the tested compounds indicated that compound **2b** formed one hydrogen bond and several arene–H bonds to the receptor, while compounds **1b** and **2a** were only bound to the receptor by one arene–H; only hydrophobic bonding was seen between compound **1a** and the receptor (see Supporting Information Figure 5).

## 3 Discussion

Liver fibrosis is one of the main causes of illness and mortality worldwide, with chronic liver damage being the most prevalent complication, which can progress to cirrhosis within 1 to 10 years.^12^ Thus, slowing fibrosis progression might be a viable treatment strategy for patients. To this end, this study aims to assess whether THQ derivatives possess an antifibrotic and hepatoprotective effect against the CCl_4_-induced hepatotoxicity and fibrosis model in rats. THQ derivatives showed no signs of toxicity, as evidenced by measurements of the different hepatic function biomarkers such as ALT, AST, STB, SA, STG, and SC, as well as kidney function biomarkers such as urea and creatinine. Additionally, the oral administration of THQ did not exhibit any decrease in the body weights of the treated rats, suggesting a safe use of THQ.

We compared the potential effects of THQ derivatives and silymarin on the liver injury model in rats. Silymarin served as a reference substance since it is a hepatoprotective and antifibrotic agent.^23^ Most studies were conducted using CCl_4_-induced acute liver damage, a well-known murine model of hepatic injury and fibrosis.^24,25^

The ALT, AST, and bilirubin are employed clinically to assess hepatocyte damage, while the determination of total protein is widely used to assess the synthetic activity of the liver.^26^ This study showed that CCl_4_ administration was associated with a significant increase in ALT and AST and a decreased level of total protein concentration. However, THQ derivative treatment resulted in a significant decrease in hepatocyte damage indices and preserved the synthetic activity of the liver.

In the present study, hematoxylin and eosin, and Masson’s Trichrome stainings were performed to assess the effect of THQ derivatives treatment on the CCl_4_-induced liver injury.^27^ CCl_4_-treated group administration led to severe steatosis, lobular inflammation, ballooning of the hepatocytes, and extensive bridging fibrosis in the liver. Histological examination revealed that silymarin was able to mitigate the extent of liver fibrosis induced by CCl_4_ to a moderate level of steatosis and focal bridging fibrosis. On the other hand, THQ derivative treatment significantly ameliorated the histopathologic findings pathognomonic of CCl_4_ damage and normalized liver architecture and portal tract; compounds **1a**, **1b**, and **2b** are more effective than compound **2a**.

To investigate whether the improvement of liver function and architecture induced by THQ derivative treatment was associated with antioxidant activity, we measured oxidative stress in the liver. We examined the level of reduced GSH and malondialdehyde (product of lipid peroxidation; MDA) as well as key biochemical markers, including the CYP2E1 metabolic enzyme.^28^ CCl_4_ administration significantly elevated the level of CYP2E1 protein expression, a member of the cytochrome P450 mixed-function oxidase system that is implicated in a variety of pathological conditions.^29^ It is considered the major isozyme involved in carbon tetrachloride bioactivation, leading to the generation of toxic intermediates and excessive amounts of ROS.^30^ There was a trend toward a reduction in CYP2E1 expression with THQ derivative treatment; however, this did not reach statistical significance, and therefore, it is difficult to implicate this reduction in the hepatoprotective mechanism of action for these compounds.

Additionally, cellular antioxidant mechanisms, such as GSH, were markedly compromised by the CCl_4_ administration.^31^ GSH is a nonenzymatic antioxidant and intracellular redox homeostasis regulator that is expressed in all cell types.^32^ Depletion of cellular GSH in the liver is thought to be a major cause of CCl_4_ toxicity.^33^ The THQ derivative treatment markedly reversed the antioxidant mechanism as compared to the CCl_4_-only group. This may have played a key role in these compounds’ hepatoprotective action against CCl_4_ toxicity. Here, the restoration of GSH levels may be due to THQ hepatoprotection against CCl_4_ toxicity. It is worth noting that compounds **1a** and **2a** had the most profound effects.

Furthermore, CCl_4_ administration significantly enhanced the oxidative stress and the concentration of MDA, a well-known product of lipid peroxidation and index of oxidative stress,^34^ an increase in which has been linked to CCl_4_-induced tissue damage. The significant rise in MDA levels in the investigated liver was reduced by the administration of THQ derivatives, with the superiority of compounds **1b**, **2a**, and **2b** over compound **1a**. This action may be related to the ability of THQ derivatives to scavenge hydroxyl radicals^35−40^ and therefore reduce lipid peroxidation.

The antioxidant activity of THQ derivatives may be attributed to their ability to scavenge ROS to some extent, possibly due to the formation of a hydrogen bond between an electronegative atom such as amine or hydroxy and a lone pair of nitrogen atoms present on an unstable free radical.^41^

To investigate whether the improvement of liver function and architecture induced by THQ derivative treatment was associated with anti-inflammatory activity, we measured the pro-inflammatory cytokines TNF-α and IL-6.^42^ In response to oxidative stress caused by CCl_4_-induced hepatotoxicity, the expression of pro-inflammatory cytokines such as TNF-α and IL-6 is induced.^35,36^ TNF-α initiates a chain reaction of cytokines that drives the inflammatory response.^43^ TNF-α is a pleiotropic proinflammatory cytokine that is rapidly generated by macrophages in response to tissue damage.^44^ It promotes oxidative metabolism and nitric oxide generation in phagocytes by stimulating the release of cytokines from macrophages. TNF-α can also cause direct cytotoxicity and has been linked to increased apoptosis.^45,46^ TNF-α is also a key player in the pathogenesis of liver fibrosis through the stimulation of Kupffer cells.^47^

TNF-α levels have been shown to increase steadily during CCl_4_ -induced hepatotoxicity,^47^ leading to fibroblast proliferation and collagen synthesis.^45,48^ However, the THQ derivative treatment, especially compounds **1b**, **2a**, and **2b**, led to a marked reduction in the TNF-α level.

CCl_4_ administration is associated with elevated IL-6 levels, a well-known pro-inflammatory factor that plays a critical role in inflammatory and immunological illnesses,^49^ IL-6 is generated by macrophages and monocytes and is vital to the liver’s local inflammatory response.^50^ THQ derivatives, especially compounds **1b**, **2a**, and **2b**, surprisingly lowered the levels of IL-6, thereby dampening the inflammatory response in the liver. These results suggest that the anti-inflammatory activity of THQ derivatives may have contributed to their hepatoprotective effect.^36,37,39,40,51−53^ Additionally, decreased ROS production by THQ culminates in the suppression of cytokine activation, thereby attenuating the expression of pro-inflammatory mediators such as TNF- α and IL-6.^54^

Since CCl_4_-induced hepatic injury is associated with extensive apoptosis,^55^ we evaluated the antiapoptotic effect of the THQ derivatives by assessing the levels of the apoptosis-related proteins (Bax and Bcl2).^56^ Apoptosis is programmed cell death that is distinguished by membrane blebbing, cell shrinkage, chromatin condensation, and nuclear fragmentation. Pro- and antiapoptotic proteins interact with one another during apoptosis to protect cellular integrity.^57^ The B-cell lymphoma-2 (Bcl-2) protein family controls mitochondrial dysfunction^58^ and includes antiapoptotic (Bcl2) and proapoptotic (Bax) effector proteins.^57,59^ Loss of the electrochemical gradient over the inner mitochondrial membrane results in the uncoupling of oxidative phosphorylation, leading to the formation of superoxide free radicals. This is a key action of Bcl-2 family proteins implicated in mitochondrial changes associated with apoptosis.^60^ In addition, cytochrome *c* (Cyt *c*) is released into the cytosol from mitochondria. Cyt *c* triggers proteolytic processing and the activation of cell death proteases known as caspases, which are essential for hepatocyte apoptosis.^61^ Overexpression of Bcl-2 prevents Cyt *c* release and caspase activation, whereas induction of Bax promotes these alterations.^62^ As a result, the ratio of Bax to Bcl2 is a crucial index of apoptotic potential. Apoptosis is induced by the upregulation of Bax and the downregulation of Bcl2.^55^

In the mitochondria of animals with CCl_4_-induced hepatic injury, the expression of Bcl-2 is downregulated, whereas the expression of Bax is upregulated, resulting in an elevated Bax/Bcl-2 ratio.^63^ Treatment with THQ derivatives caused downregulation of Bax protein and upregulation of Bcl2 protein, decreasing the Bax/Bcl2 ratio, suggesting that these compounds, especially **1b**, **2a**, and **2b**, are endowed with antiapoptotic activity.^36,40,64^

It has been shown previously that heterocyclic compounds bearing quinoline in their structure have antiapoptotic activity.^65^ Electronegative atoms impart moderate antitumor activity to compounds.^66^

Since CCl_4_ damage eventually leads to liver fibrosis, we sought to assess the potential antifibrotic effect of the tested compounds by measuring the levels of the fibrosis markers α-SMA and TGF-β. Hepatic fibrosis is an energetic process that initiates hepatocyte necrosis and then involves the activation of inflammatory cells, such as macrophages, HSC activation and proliferation, and the release of fibrogenic mediators.^3^ TGF-β family signaling pathways are master regulators of several cellular activities, such as proliferation, differentiation, migration, and cell death, that are necessary for tissue and organic homeostasis.^51^ TGF signaling is implicated in all stages of liver disease, from liver damage through inflammation and fibrosis to cirrhosis and cancer. TGF-β has cytostatic and apoptotic actions on hepatocytes, which then promote liver differentiation during embryonic development and physiological liver regeneration.^67^ TGF-β is the most significant profibrotic cytokine and promotes the production of type I procollagen.^48^ The CCl_4_-treated group showed a marked increase in TGF-β levels, possibly contributing to the CCl_4_-induced hepatic injury and fibrosis.^25,68^ THQ derivatives treatment lowered TGF-β levels in our study, with compounds **1b**, **2a**, and **2b** being more superior than **1a** in their antifibrotic effects. This suggests that the hepatoprotective effect of the compounds under investigation may be partly due to their antifibrotic activity by lowering the TGF-β level.^37,40,51^

The administration of CCl_4_ was associated with an increase in the expression of α-SMA,^69^ a distinct actin isoform found in vascular smooth muscle cells but lacking functionality in skeletal muscle fibrogenesis.^70^ However, α-SMA is important for the mobility and contraction of myofibroblasts^71^ during hepatic fibrogenesis, so they may reach the damaged liver tissue. Currently, α-SMA is believed to adjust myofibroblast contractility and signal the nucleus to control collagen formation. Thus, α-SMA can communicate with the nucleus to control collagen synthesis.^72^ Moreover, stimulation of HSCs is an essential phase in the progression of liver fibrosis.^35^ The activation of HSCs is crucial to the occurrence of liver fibrosis.^73^ Elevation of α-SMA levels acts as a phenotypic marker, indicating HSC phenotypic transition into myofibroblast-like cells. In the current study, THQ derivatives treatment reduced α-SMA expression as compared with that in the control group, with compounds **1b** and **2a** being more superior than the others, suggesting that THQ derivatives may inhibit fibrosis by blocking the activation and differentiation of HSCs into myofibroblasts.^53,74^ This reduction in α-SMA is probably a consequence of lowering TGF-β activity.^75^

The antifibrotic effect of THQ derivatives is related to the substitution pattern of the 4-phenyl group, which was found to be critical for potent TGF-β inhibitory activity. Considerable hydrophobic interactions were present in this part of the molecule for potent TGF-β inhibitory activity.^75,76^

## 4 Conclusions

This study showed that THQs were effective in lowering CCl_4_-induced acute liver damage and fibrosis in a rat model by controlling oxidative stress, lipid peroxidation, inflammation (via reduction of TNFα and IL-6 expression), and restriction of HSC activation (via regulation of TGF-β and α-SMA expression). THQ protected hepatocytes from CCl_4_-induced apoptosis in a less favorable manner by regulating the expression of the Bcl-2 family of proteins with a lower expression of proapoptotic proteins and a higher expression of antiapoptotic proteins. Histological examination revealed the beneficial effect of THQ derivatives. The results of the in silico molecular docking evaluation of the compounds against Bax, Bcl2, IL-6, TGF-β, and TNF-α were also in good agreement with the biologically obtained results. Thus, this study demonstrates that these compounds have the potential to be developed into novel therapeutics for the prevention of acute liver damage and fibrosis without deleterious effects on the kidneys.

## 5 Materials and Methods

### 5.1 Drug Synthesis

All of the reagents and solvents were purchased from commercial suppliers. Compound melting points in open glass capillaries were obtained by using the Thomas–Hoover melting point equipment. Thin-layer chromatography (TLC) using silica gel-precoated aluminum sheets (Type 60 GF254; Merck; Germany) was used to monitor the reactions and determine the purity of the chemicals employed in the study. Compounds were identified by exposing spots on TLC sheets to an ultraviolet lamp at *k* = 254 nm for few seconds. Microanalyses for purity were performed at the regional Center for Mycology and Biotechnology, El Azhar University, and the found values were within ±0.3% of the theoretical values.

#### 5.1.1 Preparation of 4-Aryl-2-hydroxy-5,6,7,8-tetrahydroquinoline-3-carbonitriles **1a** and **1b**(15)

Equimolar volumes of the suitable aldehyde (45 mmol), 4.41 g of cyclohexanone (45 mmol, 4.66 mL), 5.08 g of ethyl cyanoacetate (45 mmol, 4.78 mL), and 6.93 g of ammonium acetate (90 mmol) were heated in 25 mL of ethanol under reflux for 15 min. The mixture was then stirred for 3 h at room temperature. The majority of the solvent was removed using vacuum concentration, and the remainder was permitted to stand for 24 h, resulting in the separation of yellow crystals. These were separated by filtration, rinsed in ethanol, dried, and recrystallized in ethanol. IR (KBr) *V̅*(cm^–1^) = 3220–3222 (NH or OH associated), 2240–2250 (CN), 1695–1700 (C=O).

##### 5.1.1.1 5,6,7,8-Tetrahydro-2-hydroxy-4-phenylquinoline-3-carbonitrile (**1a**)^15^ 4H

Yield 35.7%; melting point (m.p.): 260–262 °C. ^1^H NMR (300 MHz, DMSO-*d*_6_): δ 1.63–1.68 (m, 2H, C_6_–H_2_), 1.76–1.81 (m, 2H, C_7_–H_2_), 2.61–2.66 (m, 2H, C_5_–H_2_), 2.77–2.82 (m, 2H, C_8_–H_2_), 4.21 (s, 1/, OH, D_2_O-exchangeable), 4.81 (s, 3/4H, NH, D_2_O-exchangeable), 7.32–7.45 (m, 5H, phenyl-H); ^13^C- NMR (75 MHz, DMSO-*d*_6_): δ 22.4, 27.3, 31.2, 38.4 (cyclohexyl-C), 116.65 (CN), 119.4, 121.0, 126.4, 128.7, 132.6, 133.8 152.1, 157.0, 161.8 (Ar–C).

##### 5.1.1.2 4-(4-Chlorophenyl)-2-hydroxy-5,6,7,8-tetrahydroquinoline-3-carbonitrile (**1b**)^15^

Yield 29.6%; m.p.: 296 °C. ^1^H NMR (300 MHz, DMSO-*d*_6_): 1.66–1.70 (m, 2H, C_6_–H_2_), 2.02–2.07 (m, 2H, C_7_–H_2_), 2.60–2.64 (m, 2H, C_5_–H_2_), 2.80–3.11 (m, 2H, C_8_–H_2_), 4.75 (s, 3/4H, OH, D_2_O-exchangeable), 5.14 (s, 1/4H, NH, D_2_O-exchangeable), 7.31,7.59 (2d, *J* = 7.8 Hz, each 2H, chlorophenyl-H); ^13^C NMR (75 MHz, DMSO-*d*_6_): δ 24.37, 26.29, 29.87, 31.12 (cyclohexyl-C), 116.76 (CN), 119.5, 121.6, 127.1, 129.6, 131.5, 132.6, 151.9, 157.5, 164.2 (Ar–C).

#### 5.1.2 Preparation of 2-Amino-4-aryl-5,6,7,8-tetrahydroquinoline-3-carbonitriles **2a** and **2b**(15)

In a 25 mL dry benzene mixture, equimolar proportions of the suitable aldehyde (45 mmol), 4.41 g of cyclohexanone (45 mmol), 2.97 g of malononitrile (45 mmol), and 6.93 g of ammonium acetate (90 mmol) were heated with stirring for 5 h under reflux using a Dean–Stark head. After the solvent was removed, the residue was boiled in 15 mL ethanol. The yellow crystals were separated, washed with ethanol, dried, and recrystallized from ethanol. IR (KBr) *V̅* (cm^–1^) = 3450–3300 (NH), 2222–2224 (CN), 1594, 1555, 1534, and 1486 (C=N and Ar–C=C).

##### 5.1.2.1 2-Amino-4-phenyl-5,6,7,8-Tetrahydroquinoline-3-Carbonitrile (**2a**)^15^

Yield 24.3%; m.p.: 233–235 °C. ^1^H NMR (300 MHz, DMSO-*d*_6_): δ 1.65 (m, 2H, C_6_–H_2_), 1.822 (m, 2H, C_7_–H_2_), 2.408 (t, 2H, C_5_–H_2_), 2.951 (t, 2H, C_8_–H_2_), 5.62 (s, 2H, NH_2_, D_2_O-exchangeable), and 7.395–7.552 (m, 5H, Ar–H); ^13^C NMR (75 MHz, DMSO-*d*_6_): δ 21.5, 23.2, 25.6, 27.3 (cyclohexyl-C), 112.02 (CN) and 119.45, 128.1, 130.1, 132.7, 134.4, 137.9, 150.4, 159.6, 160.9, 161.1 (Ar–C).

##### 5.1.2.2 2-Amino-4-(4-Chlorophenyl)-5,6,7,8-Tetrahydroquinoline-3-Carbonitrile (**2b**)^15^

Yield 33.7%; m.p.: 263–266 °C. ^1^H NMR (300 MHz, DMSO-*d*_6_): δ 1.64–1.68 (m, 2H, C_6_–H_2_), 1.80–1.84 (m, 2H, C_7_–H_2_), 2.62–2.67 (m, 2H, C_5_–H_2_), 2.76–2.81 (m, 2H, C_8_–H_2_), 6.43 (s, 2H, NH_2_, D_2_O-exchangeable) and 7.51,7.59 (2d, *J* = 8.0 Hz, each 2H, chlorophenyl-H); ^13^C NMR (75 MHz, DMSO-*d*_6_): δ 25.37, 26.29, 31.12, 38.22 (cyclohexyl-C), 109.5 (CN), 119.2, 120.6, 127.1, 128.6, 132.5, 132.9, 152.8, 157.0, 165.3 (Ar–C).

### 5.2 Animal Studies

Wistar albino male rats with an age of 5–7 weeks, weighing 130–200 g, were procured from the Institute of Graduate Studies & Research Animal House. The rats were maintained in well-ventilated polypropylene cages and fed standard pellets and water at a constant temperature of (24 °C ± 1). Animals were fasting but were provided unrestricted access to water overnight before the start of the experiments. All tests were executed following the faculty’s animal ethics committee’s norms and regulations (Institutional Review Board; IRB). The experimental protocol was approved by the Alexandria University-Institutional Animal Care and Use Committee (AU-IACUC)- (Approval Code. 06–2020–11–24–1–84). All authors complied with the ARRIVE guidelines, and the reporting in the manuscript follows its recommendations.

### 5.3 Drugs and Chemicals

Isoflurane 100% (Pharco-pharmaceuticals company), silymarin (South Egypt Drug Industries Co.-SEDICO), corn oil (Alpha Chemika, India), carboxy methyl cellulose sodium salt (CMC, Adwic, Egypt), carbon tetra chloride (CCl_4_, Alpha Chemika, India), formalin (Adwic, Egypt), thiobarbituric acid (TBA, Advent, India), trichloroacetic acid (TCA, Sd Fine-Chem Limited – SDFCL, Mumbai, India), ethylenediaminetetraacetic acid (EDTA, BioTech, Egypt), potassium dihydrogen phosphate (KH_2_PO_4_, Lanxess, India), disodium hydrogen phosphate (Na_2_HPO_4_, Sd Fine-Chem Limited – SDFCL, Mumbai, India), 5,5′-dithiobis (2-nitrobenzoic acid) (DTNB) (D8130-sigma aldrich, USA), 1,1,3,3-tetramethoxypropane (108383-sigma-aldrich, USA), l-glutathione reduced (G4251-sigma-aldrich, USA), and Phosphate-buffered saline (Loba Chemie, Mumbai, India).

### 5.4 Experimental Animal Design

Hundred and eight healthy adult Wistar albino rats weighing 130–200 g were divided into 14 groups (Table.2).

**Table 2:** Experimental Animal Design

main group	days	1–14	15
toxicity Study (*n* = 30)	CMC (*n* = 5)	administered a daily dose of carboxy methyl cellulose sodium salt (CMC,0.5%) in deionized water, (3 mL/kg body weight, p.o.)^77^	animals were weighed and euthanized
	SIM (*n* = 5)	administered with a daily dose of silymarin at a dose of 100 mg/kg in deionized water, p.o.^77,78^	
	A (*n* = 5)	administered with a daily dose of synthetic derivative (**1a**) at a dose of 25 mg/kg in (CMC), p.o.^77,79^	
	B (*n* = 5)	administered with a daily dose of synthetic derivative (**1b**) at a dose of 25 mg/kg in (CMC), p.o.^77,79^	
	C (*n* = 5)	administered with a daily dose of synthetic derivative (**2a**) at a dose of 25 mg/kg in (CMC), p.o.^77,79^	
	D (*n* = 5)	administered with a daily dose of synthetic derivative (**2b**) at a dose of 25 mg/kg in (CMC), p.o.^77,79^	
therapeutic model (*n* = 78)	control (*n* = 8)	administered corn oil (1 mL/kg, w/v, i.p. body weight) every 72 h^78^	
	model (*n* = 10)	injected CCl_4_ (1 mL/kg body weight, i.p. 1:1 v/v mixture of CCl_4_ and corn oil) every 72 h^78^	
	CCl_4_ + CMC (*n* = 10)	administered with CMC (same dose as the CMC group) with CCl_4_ injected (same dose as the model group)^77,78^	
	CCl_4_ + SIM (*n* = 10)	administered with silymarin (same dose as the SIM group) with CCl_4_ injected (same dose as the model group)^77,78^	
	CCl_4_ + A (*n* = 10)	administered with A (same dose as the A group) with CCl_4_ injected (same dose as the model group)^77,79^	
	CCl_4_ + B (*n* = 10)	administered with B (same dose as the B group) with CCl_4_ injected (same dose as the model group)^77,79^	
	CCl_4_ + C (*n* = 10)	administered with C (same dose as the C group) with CCl_4_ injected (same dose as the model group)^77,79^	
	CCl_4_ + D (*n* = 10)	administered with D (same dose as the D group) with CCl_4_ injected (same dose as the model group)^77,79^	

### 5.5 Dose Selection

CCl_4_ at a dose of 1 mL/kg body weight/72 h, i.p. for 14 days, was used as a previous study had demonstrated that this dose leads to a significant increase in liver function indices, oxidative stress, and levels of fibrosis markers.^78^

The dose of THQ (25 mg/kg/day, p.o.) daily for 14 days was selected based on prior findings with similar derivatives that had previously been shown to possess preventive and therapeutic properties against hepatotoxicity.^79^ The dose of silymarin (SIM, 100 mg/kg/day, p.o.) was chosen based on prior research showing hepatoprotective benefits for silymarin against various types of tissue damage and CCl_4_-induced toxicity.^78^

### 5.6 Sample Collection and Processing

Animals were weighed and euthanized 24–48 h after injection of the last dose using isoflurane (3% for 3 to 5 min).^80^ Blood was extracted through the retro-orbital puncture, allowed to clot, and then centrifuged for 10 min at 5000 rpm to extract serum. The serum was evaluated promptly for liver function enzymes. Biochemical and histological tests were conducted on the dissected liver. After cervical dislocation, the rats were euthanized, and hepatic tissue samples were collected via laparotomy. Livers were harvested and washed in ice-cold, phosphate-buffered saline. Samples were maintained at −80 °C for additional analysis of liver fibrosis, inflammatory markers, oxidative stress, and apoptosis.

### 5.7 Assessment of the Hepatoprotective Activity

#### 5.7.1 Serum Biochemical Analysis

Several liver biomarkers were determined using standard clinical chemistry assays via commercial kits. Serum levels of aspartate transaminase (AST), alanine transaminase (ALT), serum total bilirubin (STB), and serum albumin (SA). Serum triglyceride (STG), serum cholesterol (SC) levels, and kidney biomarkers, such as serum urea and serum creatinine, were measured.

#### 5.7.2 Histopathological Examination

Liver specimens were preserved in 10% formalin-buffered saline, dried, and immediately immersed in firm paraffin. These specimens were then sectioned into 5 μm segments and stained with hematoxylin and eosin and Masson’s trichrome stain for photomicroscopy examination using the software Leica Microsystems (Switzerland) for evaluating the extent of tissue damage and deposition of the collagen.

#### 5.7.3 Hepatic Tissue Analysis

##### 5.7.3.1 Assessment of Oxidative Stress Markers

Levels of reduced GSH were analyzed, as described by Richardson and Murphy,^81^ and levels of malondialdehyde (MDA) were determined using thiobarbituric acid, as described by Ohkawa et al., (1979).^82^

##### 5.7.3.2 Enzyme-Linked Immunosorbent Assay

Tumor necrosis factor alpha (TNF-α, San Diego; Catalog no. MBS2507393), Transforming growth factor beta (TGF- β, San Diego, USA; Catalog no. MBS824788), Cytochrome P450 2E1 (CYP2E1, Houston, USA; Catalog no. CSB E09782r ), alpha-smooth muscle actin (α-SMA, Houston, USA; Catalog no. CSB E14027r ), and interleukin 6 (IL-6, Houston, USA; Catalog no. CSB E04640r ) were measured by enzyme-linked immunosorbent assay (ELISA) kit.

##### 5.7.3.3 Reverse Transcription Polymerase Chain Reaction

The relative expression of Bax and Bcl2, apoptotic progression markers, was measured using RT-PCR. Total RNA was purified from liver tissue samples using a TriFast reagent. For quality and yield determination, the ratio of absorbance at 260 and 280 nm was assessed using the NanoDrop Spectrophotometer. In a two-step RT-PCR setup, total RNA was reverse-transcribed into single-stranded complementary DNA using the QuantiTect Reverse Transcription Kit (Qiagen, USA) and a random primer hexamer. Rotor-Gene Q (Qiagen, USA) was used to examine the mRNA of genes, with β-actin serving as a housekeeping gene. cDNA amplicons were amplified using Applied Biosystems TaqMan Universal PCR Master Mix (Applied Biosystems, Foster City, CA, USA) and specific primers: Bcl-2 gene; forward; 5′-GGATCCAGGAT AACGGAGGC-3′, reverse; 5′-CCAGATAGG CACCCAGGGT-3′, Bax gene; forward; 5′-CTTTTGCTTCAG GGTTTCATCC-3′, reverse; 5′-TTGAGACACT CGCTCAGCTTCT-3′, β-actin (House-Keeping Gene); forward; 5′-CCTGGCACCCA GCACAA-3′, reverse; 5′-GCCGATCCAC ACGGAGTACT-3′. The relative expression of the target gene was computed using the 2^–ΔΔct^ method.^83^

### 5.8 Docking Study

Molecular Operating Environment (MOE Dock 2015) software (Chemical Computing Group, Montreal, QC) was used to perform computer-assisted simulation docking experiments using an MMFF94X force field.

#### 5.8.1 Docking Protocol

Crystal structures of Bax, Bcl2, IL-6, TGF-β, and TNF-α were obtained from the Protein Data Bank (PDB ID: 6EB6, 5WHH, 1ALU, 1TGJ, and 2AZ5, respectively) and used in the docking simulations. The ligand molecules were constructed using the builder molecule and were energy-minimized. Ligands were docked within the active site by using the MOE Dock. The lowest energy conformation was selected, and the ligand interactions such as hydrogen bonding, arene–H, and arene–arene interactions, together with other hydrophobic interactions with the receptors, were recorded.

### 5.9 Statistical Analysis

Statistical analysis was performed using Prism 8.0.2 (263) software (GraphPad, La Jolla, CA). Differences between groups for parametric data were analyzed using a one-way analysis of variance with Tukey’s posthoc test, and nonparametric data were analyzed using Kruskal–Wallis’s test with the appropriate Dunn’s multiple comparisons test.

## Data Availability

The data sets generated during and/or analyzed during the current study are available in the published article and its Supporting Information.

## Supporting Information Available

Docking analysis using crystal structures for the reference (Silymarin) and test compounds **1a**, **1b**, **2a**, and **2b** within the Bax, Bcl2, IL-6, TGF-β, and TNF-α active sites (PDF)

## Accession Codes

Crystal structures of Bax, Bcl2, IL-6, TGF-β, and TNF-α were obtained from the Protein Data Bank (PDB ID: 6EB6, 5WHH, 1ALU, 1TGJ, and 2AZ5, respectively) and used in the docking simulations.

## Notes

The study was executed following the faculty’s animal ethics committee’s norms and regulations (Institutional Review Board; IRB) and Alexandria University’s Institutional Animal Care and Use Committee (AU-IACUC) (Approval Code. 06–2020–11–24–1–84).

## Abbreviations

THQ: tetrahydroquinoline
GSH: glutathione
MDA: malondialdehyde
TNF-α: tumor necrosis factor-alpha
TGF-β: transforming growth factor-beta
α-SMA: alpha-smooth muscle actin
IL-6: interleukin 6
CCl_4_: carbon tetrachloride
ECM: extracellular matrix
HSC: hepatic stellate cell
AChEI: acetylcholinesterase inhibitor
THAs: tetrahydroacridines
DMF: dimethylformamide
ALT: alanine transaminase
m.p.: melting point
IRB: Institutional Review Board
SEDICO: South Egypt Drug Industries Co
CMC: carboxy methyl cellulose
TBA: thiobarbituric acid
TCA: trichloroacetic acid
EDTA: ethylenediaminetetraacetic acid
KH_2_PO_4_: potassium dihydrogen phosphate
Na_2_HPO_4_: disodium hydrogen phosphate
DTNB: 5,5′-dithiobis (2-nitrobenzoic acid)
AST: aspartate transaminase
H&E staining kit: hematoxylin and eosin
i.p.: intraperitoneal
p.o.: orally
STB: serum total bilirubin
SA: serum albumin
STG: serum triglycerides
SC: serum cholesterol
ELISA: enzyme-linked immunosorbent assay
qPCR: real-time quantitative polymerase chain reaction
SEM: standard error of mean
Bcl-2: B-cell lymphoma-2
Cyt *c*: cytochrome *c*
ROS: reactive oxygen species
