# Supplementary material for: Structure Modification Converts the Hepatotoxic Tacrine into Novel Hepatoprotective Analogs
Source: ACS Omega. 2024 Jan 2;9(2):2491–503. doi: 10.1021/acsomega.3c07126 (PMC10795119; doi:10.1021/acsomega.3c07126)
Supplement: Supplementary file 1 — ao3c07126_si_001.pdf [file ao3c07126_si_001.pdf]

Structure modification converts the hepatotoxic tacrine into novel hepatoprotective analogs

Amani A. Sorour[1], Rania G. Aly[2], Hanan M. Ragab[3], Ahmed Wahid[1]*

[1]Department of Pharmaceutical Biochemistry, Faculty of Pharmacy, Alexandria University, Alexandria, Egypt, 21521.

[2]Department of Pathology, Faculty of Medicine, Alexandria University, Alexandria, Egypt, 21521.

[3]Department of Pharmaceutical Chemistry, Faculty of Pharmacy, Alexandria University, Alexandria, Egypt, 21521.

***Corresponding author**

Ahmed Wahid

Department of Pharmaceutical Biochemistry, Faculty of Pharmacy, Alexandria University, Alexandria, Egypt, 21521.

Tel.: +201125566987

Email: ahmed.wahid@alexu.edu.eg

Table of Contents

Supporting information Table S1 3
Supporting information Table S2 3
Supporting information Table S3 4
Supporting information Table S4 4
Supporting information Table S5 5
Supporting information Figure S1 6
Supporting information Figure S2 7
Supporting information Figure S3 8
Supporting information Figure S4 9
Supporting information Figure S5 10

Supporting information Table S1

Table S1: Male albino rats' body weight through the 14 days of the experiment

	**CMC**	**SIM**	**A**	**B**	**C**	**D**
**Body weight (Before) (grams)**	116.8±4.97	115.4±2.06	120.4±5.27	128.6±3.33	131.02±2.62	118.6±2.5
**Body weight (After) (grams)**	178.08±5.42	177.82±3.88	175.74±7.57	181.74±6.12	184.24±7.5	176.7±4.33

Values represent mean ± SEM (n = 5).

Supporting information Table S2

Table S2: Histological scoring of steatohepatitis and staging of fibrosis

	**Control**	**Model**	**CCl_4+CMC**	**CCl_4+SIM**	**CCl_4+A**	**CCl_4+B**	**CCl_4+C**	**CCl_4+D**
**Steatosis**	0	3±0.2$^{\#\#\#}$	3±0.2$^{\#\#\#\#}$	2±0.2$^{\#}$	1±0.4^{*++}	0±0.2^{***+++}	2±0.2$^{\#}$	1±0.2^{**++}
**Inflammation**	0	2±0.4	1±0.3	1±0.2	0±0.2	1±0.2	0±0.2	0±0.2
**Ballooning**	0	1±0.2$^{\#}$	1±0.2$^{\#}$	1±0.2	0±0.2	0±0.2	1±0.2	1±0.2
**Fibrosis**	0	2±0.2$^{\#\#\#}$	2±0.2$^{\#\#}$	1±0.2$^{\#\#}$	0±0.2^{**++}	0±0.2^{**++}	1±0.4^{*+}	0±0.2^{**++}

Values represent mean ± SEM (n = 5). P-value: 0.0332(*),0.0021(**),0.0002(***),>0.0001(****). Statistical analysis was performed using the Kruskal-Wallis test followed by Dunn's multiple comparisons test, # Significantly different from the normal control group, * Significantly different from the Negative control group, + Significantly different from CCl_4+CMC control group

Supporting information Table S3

Table S3: Effect of synthetic drugs on Oxidative stress markers such as MDA, GSH, and CYP 2E1 protein expression values by ELISA analysis.

	Control	Model	CCl_4+CMC	CCl_4+SIM	CCl_4+A	CCl_4+B	CCl_4+C	CCl_4+D
**MDA** **(nmole/ml)**	6.02±0.08	8.65±0.59$^{\#\#\#\#}$	8.04±0.44$^{\#\#}$	8.08±0.51$^{\#\#}$	6.17±0.16^{***++}	5.77±0.19$^{****+++}$	5.37±0.15$^{****++++}$	5.57±0.06$^{****+++}$
**GSH** **(microgram/ml)**	92.23±4.10	58.23±0.85$^{\#\#\#\#}$	65.80±0.76$^{\#\#\#\#}$	65.97±2.62$^{\#\#\#\#}$	83.14±1.38$^{****+++}$	81.63±1.36^{****++}	96.73±4.31$^{****++++}$	81.59±0.12^{****++}
**CYP 2E1** **(ng/mg protein)**	0.53±0.03	0.79±0.05$^{\#}$	---	0.35±0.04***	0.69±0.06	0.59±0.03	0.55±0.05	0.72±0.08

Values represent mean ± SEM (n=3-5).P-value: 0.0332(*),0.0021(**),0.0002(***),>0.0001(****).(****).Statistical analysis was performed using one-way ANOVA followed by Tukey-Kramer multiple comparisons test, # Significantly different from the normal control group, * Significantly different from the Negative control group, + Significantly different from CCl4+CMC control group

Supporting information Table S4

Table S4: M-RNA expression by RT-PCR analysis of (D); Bax, (E); Bcl2, and (F); Bax/Bcl2 ratio values.

	Control	Model	CCl_4+SIM	CCl_4+A	CCl_4+B	CCl_4+C	CCl_4+D
**Bax** **2^-ΔΔCt**	0.3±0.0	1.5±0.1$^{\#\#\#\#}$	1.2±0.0$^{\#\#\#}$	1.3±0.2$^{\#\#\#}$	1.0±0.1$^{\#\#*}$	0.4±0.0***	0.7±0.1***
**Bcl2** **2^-ΔΔCt**	1.95±0.1	0.45±0.1$^{\#\#\#\#}$	0.75±0.1$^{\#\#\#\#}$	0.85±0.1$^{\#\#\#\#*}$	1.2±0.1$^{\#\#\#***}$	1.65±0.0****	1.35±0.0$^{\#\#***}$
**Bax/Bcl2**	0.15±0.00	3.28±0.48$^{\#\#\#}$	1.54±0.04$^{\#**}$	1.49±0.26$^{\#**}$	0.79±0.02***	0.24±0.01***	0.48±0.02***

Values represent mean ± SEM (n=2). P-value: 0.0332(*),0.0021(**),0.0002(***),>0.0001(****). Statistical analysis was performed using one-way ANOVA followed by the Tukey-Kramer multiple comparisons test. # Significantly different from the normal control group, * Significantly different from the model group.

Supporting information Table S5

Table S5: Proinflammatory, and profibrogenic cytokines level of expression through fibrosis and after treatment: TNF- α, TGF- β, α -SMA, IL-6 protein expression levels by ELISA analysis

	**Control**	**Model**	**CCl_4+SIM**	**CCl_4+A**	**CCl_4+B**	**CCl_4+C**	**CCl_4+D**
**TNF- α** (pg/100 mg protein)	47.71±0.5	95.27±0.6$^{\#\#\#\#}$	69.67±0.4$^{\#\#\#\#****}$	78.32±0.4$^{\#\#\#\#****}$	68.49±0.3$^{\#\#\#\#****}$	55.27±0.8$^{\#\#\#\#****}$	59.12±0.2$^{\#\#\#\#****}$
**TGF- β** (ng/mg protein)	37.77±0.3	66.30±0.8$^{\#\#\#\#}$	48.80±1.1$^{\#\#\#\#****}$	66.25±0.2$^{\#\#\#\#}$	54.23±0.9$^{\#\#\#\#****}$	42.83±0.7$^{\#\#****}$	45.13±1.2$^{\#\#\#****}$
**α -SMA** (ng/mg protein)	11±2.0	41.5±1.5$^{\#\#\#}$	9.5±1.5****	35.5±2.5$^{\#\#\#}$	29.5±1.5$^{\#\#*}$	25.5±1.5$^{\#**}$	34.5±2.5$^{\#\#\#}$
**IL-6** (pg/mg protein)	50±1.0	76±4.0$^{\#}$	30±2.0**	57±9.0	50±3.0*	49±4.0*	60.5±4.5

Values represent mean ± SEM (n = 2-3). P-value: 0.0332(*),0.0021(**),0.0002(***),>0.0001(****). Statistical analysis was performed using one-way ANOVA followed by the Tukey-Kramer multiple comparisons test. # Significantly different from the normal control group, * Significantly different from the model group

Supporting information Figure S1

Docking analysis using crystal structures for the reference (Silymarin) and test compounds **1a, 1b**, **2a,** and **2b** within the **Bax** active site.

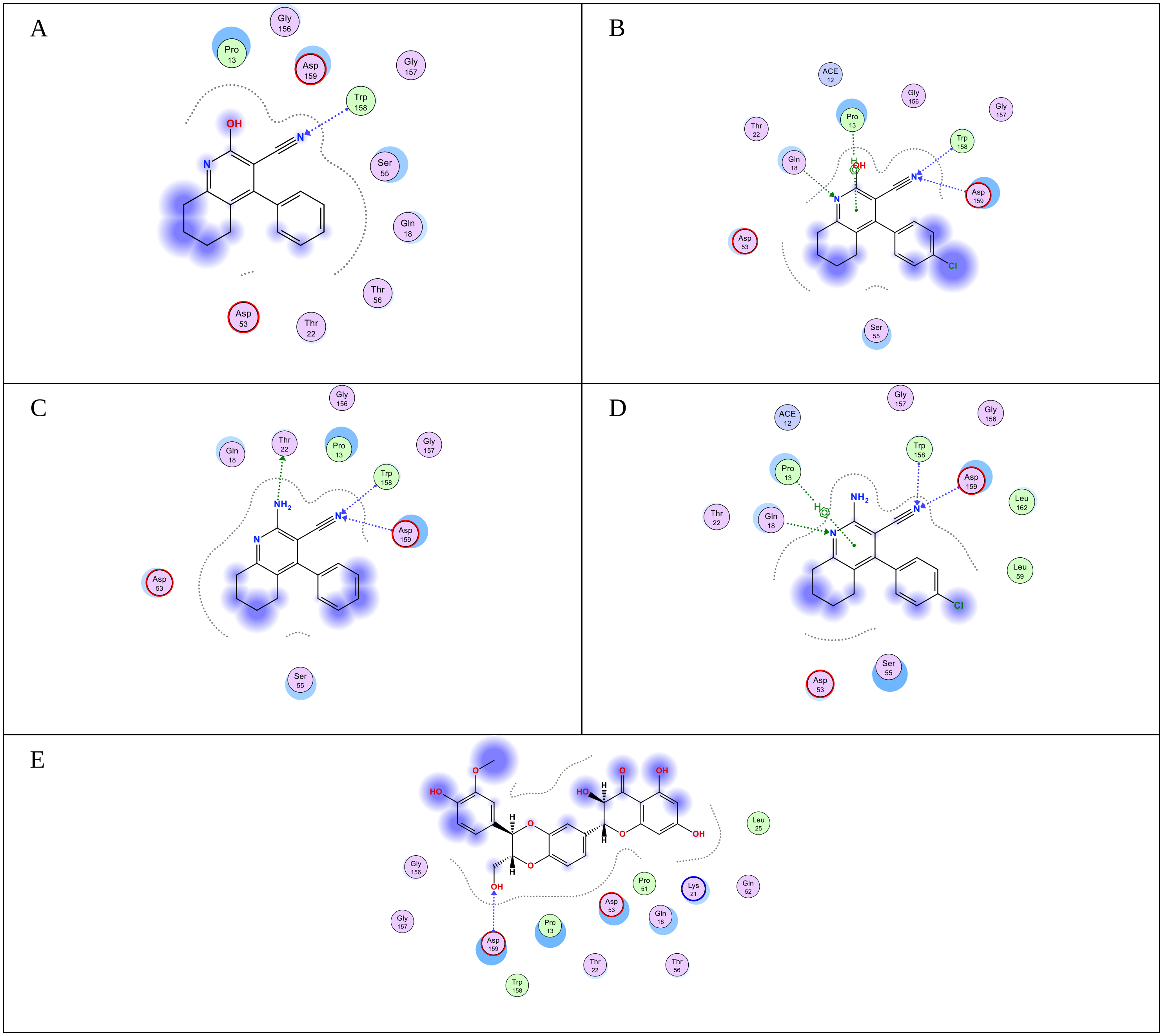

Figure S1: 2D Binding mode of the reference (Silymarin) and test compounds 1a, 1b, 2a, and 2b in the binding site of Bax receptor (PDB ID: 6eb6) using MOE software. Ligands were docked within the Bax active site using the MOE Dock, showing ligand interactions such as hydrogen bonding, arene-H, and arene-arene interactions together with other hydrophobic interactions with the receptors. (A) Compound 1a. (B) Compound 1b. (C) Compound 2a. (D) Compound 2b. (E) Silymarin.

Supporting information Figure S2

Docking analysis using crystal structures for the reference (Silymarin) and test compounds **1a, 1b**, **2a,** and **2b** within the **Bcl2** active site.

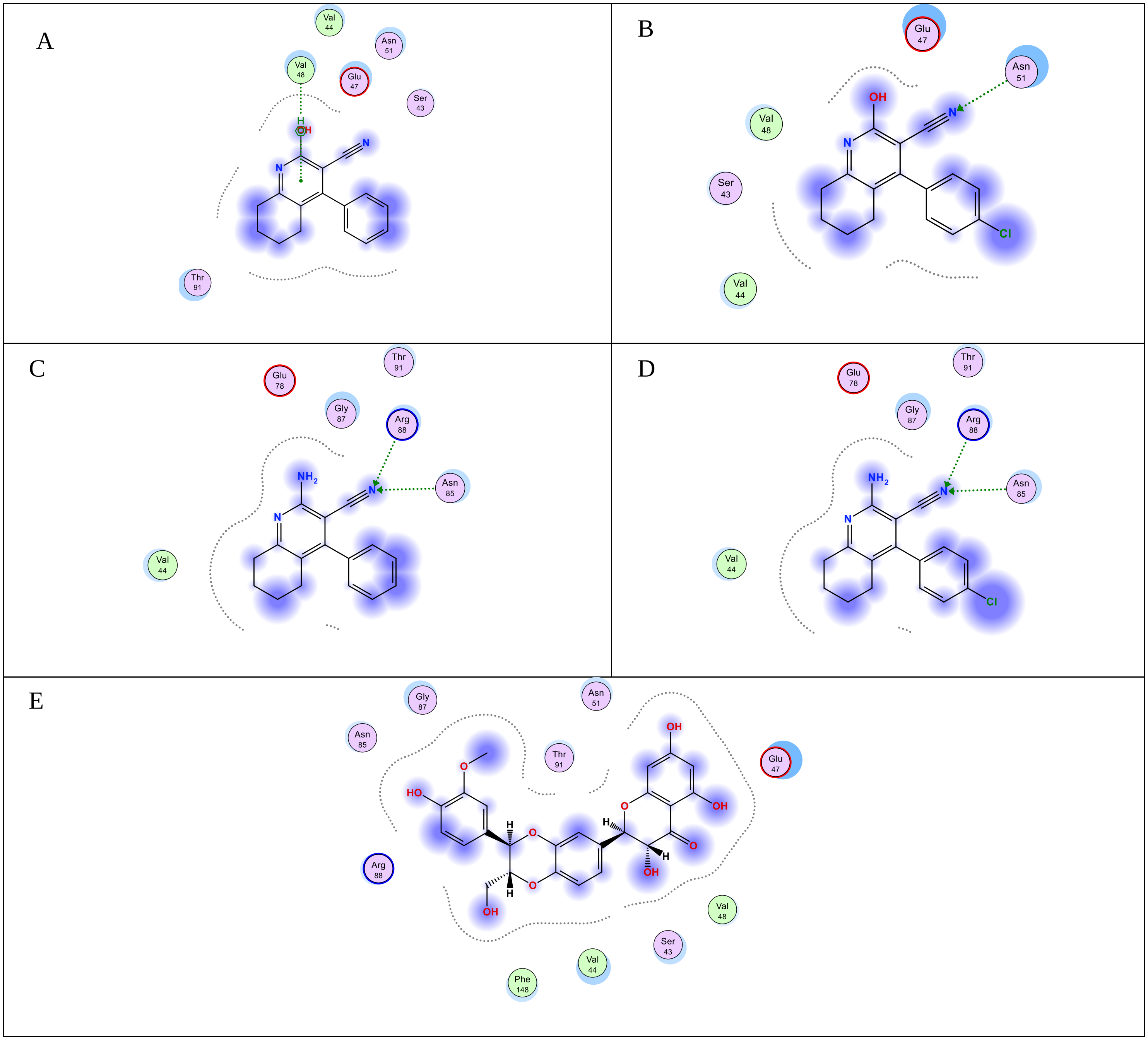

Figure S2: 2D Binding mode of the reference (Silymarin) and test compounds 1a, 1b, 2a, and 2b in the binding site of Bcl2 receptor (PDB ID: 5whh) using MOE software. Ligands were docked within the Bcl2 active site using the MOE Dock, showing ligand interactions such as hydrogen bonding, arene-H, and arene-arene interactions together with other hydrophobic interactions with the receptors. (A) Compound 1a. (B) Compound 1b. (C) Compound 2a. (D) Compound 2b. (E) Silymarin.

Supporting information Figure S3

Docking analysis using crystal structures for the reference (Silymarin) and test compounds **1a, 1b**, **2a,** and **2b** within the **IL-6** active site.

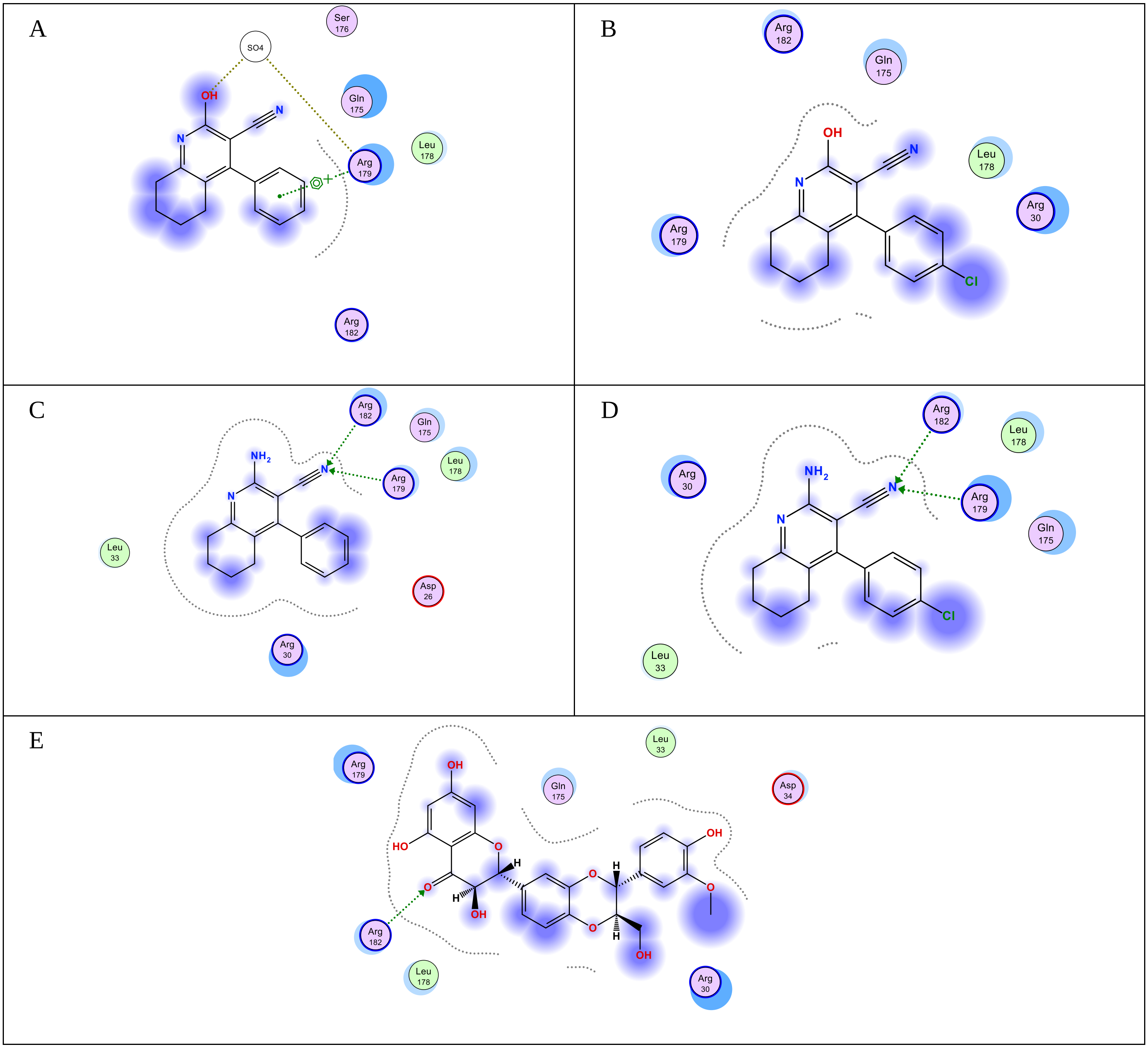

Figure S3: 2D Binding mode of the reference (Silymarin) and test compounds 1a, 1b, 2a, and 2b in the binding site of IL-6 receptor (PDB ID: 1alu) using MOE software. Ligands were docked within the IL-6 active site using the MOE Dock, showing ligand interactions such as hydrogen bonding, arene-H, and arene-arene interactions together with other hydrophobic interactions with the receptors. (A) Compound 1a. (B) Compound 1b. (C) Compound 2a. (D) Compound 2b. (E) Silymarin.

Supporting information Figure S4

Docking analysis using crystal structures for the reference (Silymarin) and test compounds **1a, 1b**, **2a,** and **2b** within the **TGF-β** active site.

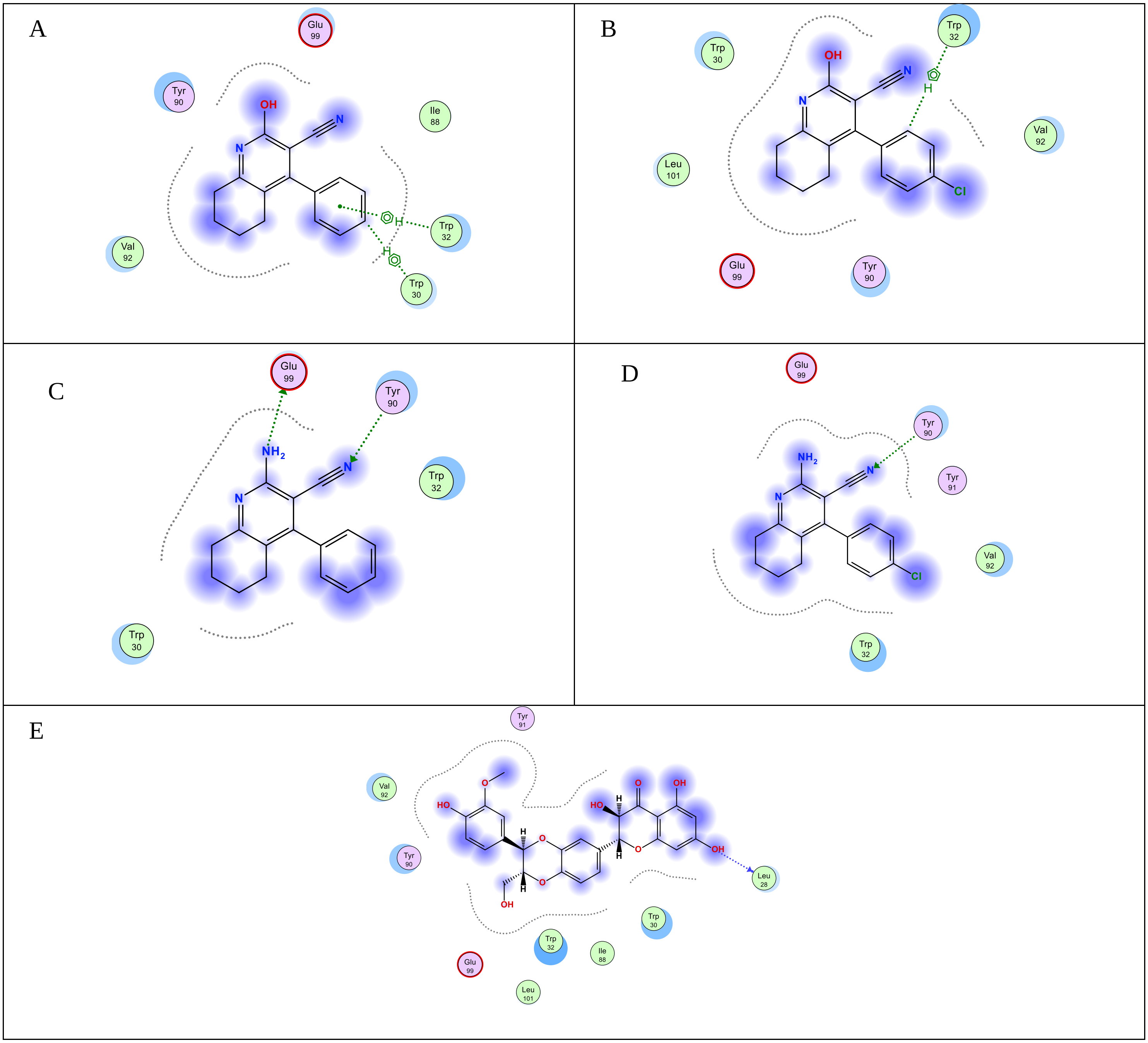

Figure S4: 2D Binding mode of the reference (Silymarin) and test compounds 1a, 1b, 2a, and 2b in the binding site of TGF-β receptor (PDB ID: 1tgj) using MOE software. Ligands were docked within the TGF-β active site using the MOE Dock, showing ligand interactions such as hydrogen bonding, arene-H, and arene-arene interactions together with other hydrophobic interactions with the receptors. (A) Compound 1a. (B) Compound 1b. (C) Compound 2a. (D) Compound 2b. (E) Silymarin.

Supporting information Figure S5

Docking analysis using crystal structures for the reference (Silymarin) and test compounds **1a, 1b**, **2a,** and **2b** within the **TNF-α** active site.

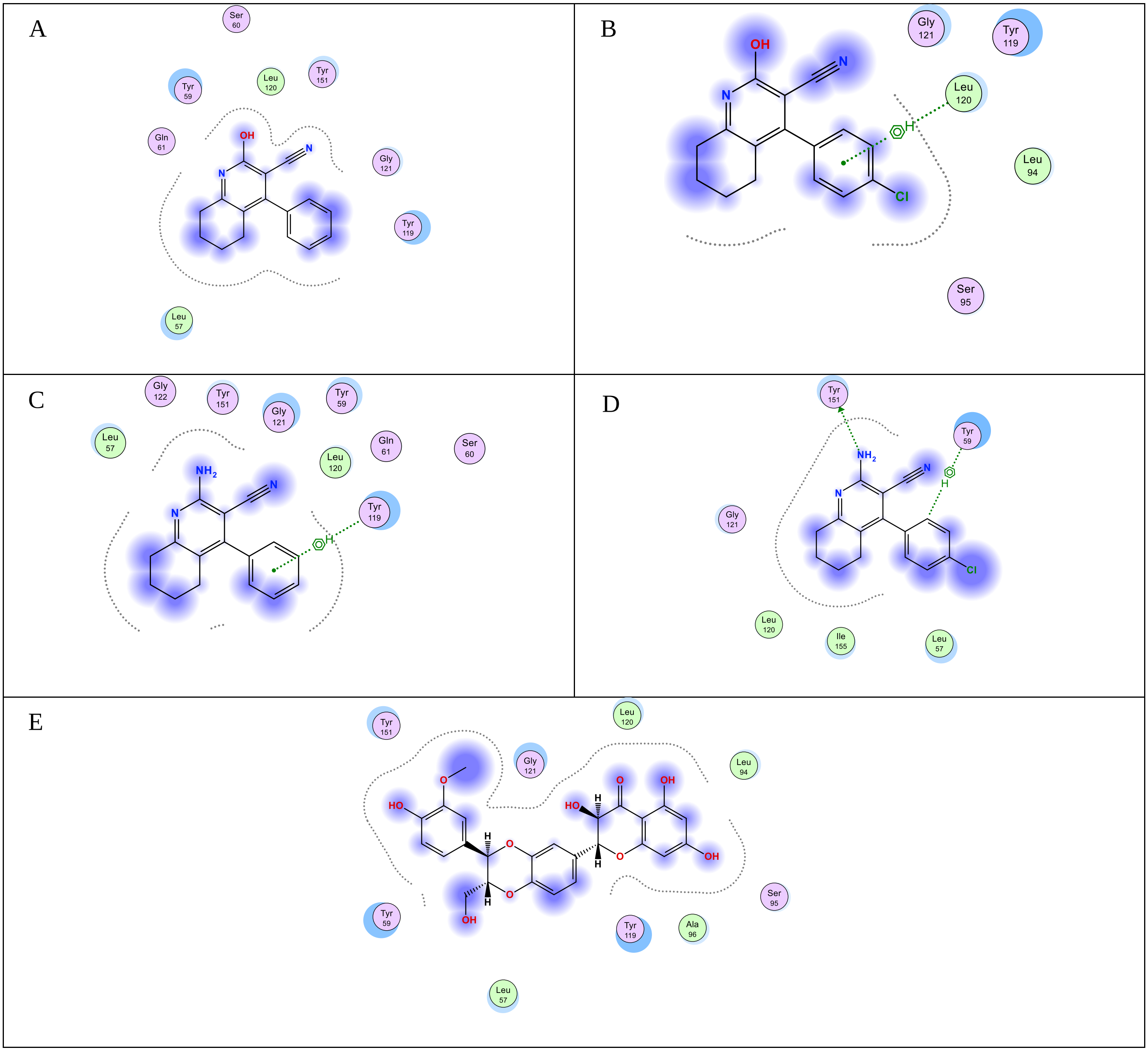

Figure S5: 2D Binding mode of the reference (Silymarin) and test compounds 1a, 1b, 2a, and 2b in the binding site of TNF-α receptor (PDB ID: 2az5) using MOE software. Ligands were docked within the TNF-α active site using the MOE Dock, showing ligand interactions such as hydrogen bonding, arene-H, and arene-arene interactions together with other hydrophobic interactions with the receptors. (A) Compound 1a. (B) Compound 1b. (C) Compound 2a. (D) Compound 2b. (E) Silymarin.
